# Risk factors for SARS-CoV-2 infection: a case-control study in college students after vaccination

**DOI:** 10.1093/eurpub/ckac129.045

**Published:** 2022-10-25

**Authors:** E Renzi, V Baccolini, A Covelli, G Migliara, A Massimi, C De Vito, C Marzuillo, P Villari

**Affiliations:** Public Health and Infectious Diseases, Sapienza University of Rome, Rome, Italy

## Abstract

**Background:**

Within the SARS-CoV-2 screening campaign offered through RT-PCR test by Sapienza University of Rome, we conducted a case-control study to identify the risk factors for the acquisition of SARS-CoV-2 infection among university students.

**Methods:**

Positive students identified through the SARS-CoV-2 screening campaign (September 2021 - February 2022) were enrolled as cases and matched to two randomly selected students who tested negative on the same day. The interview questionnaire consisted of 39 questions investigating exposure to modifiable and nonmodifiable risk factors for SARS-CoV-2 in the two weeks before testing. A multivariable conditional logistic regression model was constructed to identify predictors of SARS-CoV-2 infection. Adjusted odds ratio (aOR) and 95% CI were calculated.

**Results:**

Out of 8.730 tests for SARS-CoV-2, 173 students tested positive (2.0%), of which 122 were included in the case-control study (response rate: 70.5%). Most students were female (73.2%), with a mean age of 23.3 years (SD ± 3.6), vaccinated for SARS-CoV-2 (97.8%) and enrolled in non-health faculty (56.8%). At the multivariable analysis, significant positive associations were found with having had contact with a person who tested positive for SARS-CoV-2 (aOR: 3.04, 95% CI: 1.59-5.82) or having been to a disco/nightclub (aOR: 5.37, 95% CI: 2.00-14.38). Instead, being vaccinated against SARS-CoV-2 (aOR: 0.13, 95% CI: 0.01-0.93), having a valid EU COVID digital certificate (aOR: 0.06, 95% CI: 0.01-0.30) and attending lectures in-person (aOR: 0.35, 95% CI: 0.17-0.70) were negatively predictors. No association was found for sex, age, health faculty students, use of public transportation, attendance at restaurants or gyms.

**Conclusions:**

The results highlight how anti-COVID-19 vaccinations and the reasons for students to obtain an EU COVID digital certificate may prevent students from getting infected. In addition, university environment seems to be safe for students.

**Key messages:**

• Promoting SARS-CoV-2 vaccination adherence in the college-age population is crucial to limiting the SARS-CoV-2 spread.

• Attending in-person educational activities in regulated settings (e.g., low occupancy, mask use) may not be a risk factor for COVID-19 infection.

